# Early-Onset and Syndromic Pediatric Epilepsy in Kazakhstan: Clinical, Molecular, and Phenotypic Spectrum

**DOI:** 10.3390/jcm15103625

**Published:** 2026-05-08

**Authors:** Mirgul Bayanova, Lyazzat Nazarova, Askhat Zhakupov, Dias Malik, Aidana Gabdulkayum, Zhanel Mirmanova, Nazerke Satvaldina, Saule Rakhimova, Ainur Akilzhanova, Dauren Yerezhepov, Aidos Bolatov

**Affiliations:** 1 Clinical-Academical Department of Laboratory Medicine, Pathology, and Genetics, Corporate Fund “University Medical Center”, 010000 Astana, Kazakhstan; mirgul.bayanova@umc.org.kz (M.B.); layzzat.nazarova@umc.org.kz (L.N.); md.zhakupovaskhat@gmail.com (A.Z.); dias.malik@umc.org.kz (D.M.); 2 Laboratory of Genomic and Personalized Medicine, Center for Life Sciences, National Laboratory Astana, Nazarbayev University, 010000 Astana, Kazakhstan; aidana.gabdulkayum@nu.edu.kz (A.G.); nazerke.satvaldina@nu.edu.kz (N.S.); saule.rakhimova@nu.edu.kz (S.R.); akilzhanova@nu.edu.kz (A.A.);; 3 Shenzhen University Medical School, Shenzhen University, Shenzhen 518060, China

**Keywords:** epilepsy, genetics, childhood, epileptic encephalopathy, next-generation sequencing, early-onset epilepsy, syndromic epilepsy

## Abstract

**Background:** Pediatric epilepsy is a clinically and genetically heterogeneous group of disorders, particularly in early-onset and syndromic presentations. Data integrating molecular findings with detailed clinical phenotyping remain limited in Kazakhstan and Central Asia. **Methods:** We conducted a retrospective, single-center, clinically selected, referral-based case series study of 31 pediatric patients evaluated at a tertiary center in Kazakhstan for epilepsy or epilepsy-associated neurodevelopmental disorders and found to have clinically relevant or potentially relevant genetic findings. Clinical records were reviewed for demographics, age at seizure onset, seizure semiology, developmental profile, EEG, MRI, extra-neurological features, treatment response, and family history. Variants were interpreted using ACMG-based criteria, and inheritance/segregation data were incorporated where available. **Results:** The cohort included 15 males and 16 females. Median seizure onset was 5.0 months (IQR 2.0–13.0), and 22/31 patients (71.0%) presented within the first year of life. Developmental delay/intellectual impairment was observed in 20/31 cases (64.5%), speech delay in 17/31 (54.8%), motor delay in 15/31 (48.4%), and hypotonia in 12/31 (38.7%). Variants were identified in 23 genes, including several variants with limited or no prior support in ClinVar or peer-reviewed reports. Ion channelopathies were the largest mechanistic group (12/31, 38.7%), followed by mitochondrial/metabolic disorders (6/31, 19.4%), mTOR pathway disorders (5/31, 16.1%), and neurodevelopmental/chromatin/transcriptional disorders (4/31, 12.9%). *SCN1A* was the most recurrent gene (8/31, 25.8%) and showed a broad phenotypic continuum from Dravet-compatible to non-Dravet presentations. EEG and MRI abnormalities were common and often syndromic in pattern. Treatment response was frequently partial or poor, although selected genotype-linked treatment observations were noted in *PRRT2* (carbamazepine), *PNPO* (pyridoxine), and one *SCN1A* case (stiripentol). **Conclusions:** This study expands the clinicogenetic characterization of pediatric epilepsy cases with clinically relevant or potentially relevant genetic findings in Kazakhstan and highlights the value of integrated molecular, phenotypic, and segregation analysis in underrepresented populations.

## 1. Introduction

Pediatric epilepsy comprises a broad and overlapping spectrum of seizure syndromes, from self-limited epilepsies to developmental and epileptic encephalopathies, and is often accompanied by intellectual, behavioral, motor, and autism-related comorbidities, reflecting substantial underlying genetic heterogeneity rather than a single disease entity [1,2]. Monogenic causes are particularly important in neonatal- and infantile-onset epilepsies and in developmental/epileptic encephalopathies, where early seizure onset, developmental impairment, and drug resistance increase the likelihood of an identifiable genetic diagnosis [3]. Over the past decade, next-generation sequencing has transformed the diagnostic evaluation of childhood epilepsy by expanding the number of recognized epilepsy genes and showing that similar phenotypes may result from the disruption of diverse pathways, including ion channel, synaptic, metabolic, signaling, and transcriptional mechanisms, thereby refining clinicogenetic classification beyond syndrome-based approaches [4,5]. Consistent with this, systematic reviews and cohort studies have demonstrated meaningful diagnostic yield for sequencing-based testing, particularly for exome or genome sequencing in children with early-onset seizures, developmental delay, or refractory epilepsy [6,7,8]. Beyond establishing etiology, genomic testing increasingly informs prognosis, recurrence risk counseling, surveillance for extra-neurological manifestations, and, in selected disorders, treatment decisions [9,10]. Accordingly, current evidence supports approaching pediatric epilepsy, especially early-onset and developmentally complicated forms, as a genetically heterogeneous group of disorders for which sequencing technologies are central to modern diagnostic, clinical and phenotypic stratification [11,12,13].

Molecular diagnosis has become clinically important in pediatric epilepsy because it moves the evaluation beyond syndromic description toward an etiologic diagnosis that can explain both seizures and accompanying neurodevelopmental features [4,14,15]. Distinguishing among major biological categories (such as ion channel disorders, mTOR pathway disorders, metabolic epilepsies, and syndromic neurodevelopmental conditions) has practical value because these groups differ in associated comorbidities, expected disease course, and surveillance needs [16,17,18]. In selected disorders, genetic diagnosis may also inform treatment choice by identifying candidates for mechanism-based therapy or by helping clinicians avoid less suitable medications, a concept increasingly framed within precision medicine for epilepsy [19,20,21]. Studies of clinical utility further show that exome-based diagnoses can influence management after testing, including medication adjustments, referrals, and syndrome-specific follow-up, rather than serving only as a descriptive laboratory endpoint [10,15]. Molecular diagnosis also improves family assessment by refining recurrence risk counseling and supporting targeted cascade evaluation when a pathogenic or likely pathogenic variant is identified, and current evidence-based guidance now recommends genetic testing together with counseling for unexplained epilepsies [22,23]. Accordingly, in contemporary pediatric epilepsy practice, genetic testing is increasingly relevant not only for establishing etiology, but also for stratifying disease mechanisms, contextualizing treatment opportunities, and informing counseling for affected families [24].

Despite rapid progress in epilepsy genomics, the published evidence base remains concentrated in selected and relatively well-studied populations, while broader neurodevelopmental genetics research continues to show substantial ancestry imbalance and underrepresentation of non-European groups, limiting the generalizability of variant interpretation and precision-medicine frameworks [25,26,27]. In Kazakhstan, recent epilepsy research has increasingly characterized the national and health system burden of epilepsy, but has focused mainly on epidemiology, access to care, status epilepticus, and surgical services rather than detailed pediatric clinicogenetic phenotyping. A nationwide administrative study using the Unified National Electronic Health System reported a marked increase in epilepsy incidence and prevalence between 2014 and 2020, with a substantial disability burden and higher mortality among children and older adults [28]. Earlier work from southern Kazakhstan highlighted urban–rural differences and treatment gaps in adult epilepsy care [29]. More recent national data on status epilepticus showed a threefold increase in age-standardized incidence between 2014 and 2023 and emphasized the need to improve emergency treatment pathways [30]. Studies of epilepsy surgery in Kazakhstan have shown that resective surgery can achieve outcomes comparable with international benchmarks, but also identified barriers related to referral pathways, geography, social factors, and access to advanced presurgical evaluation [31,32]. In the pediatric setting, regional healthcare reports have demonstrated substantial disparities in epilepsy prevalence, pediatric neurologist availability, diagnostic resources, and antiseizure medication provision across Kazakhstan [33]. Together, these studies define the epidemiologic, service delivery, and policy context of epilepsy care in Kazakhstan, but they do not provide a deeply phenotyped pediatric cohort integrating molecular findings with seizure semiology, neurodevelopmental profile, EEG, MRI, treatment observations, and family-based segregation data. Genomic studies from Kazakhstan are emerging but remain limited in number and scale. These include a position paper identifying genomic investigation of epileptic encephalopathies as an urgent priority, whole-genome sequencing cohorts of early-onset pediatric epilepsy, and single-case genomic reports describing rare or novel variants [34,35,36]. Collectively, these reports demonstrate the feasibility and value of epilepsy genomics in Kazakhstan, but they also show that local evidence is still dominated by small cohorts and individual reports, leaving a need for datasets that integrate variant interpretation with detailed seizure semiology, neurodevelopmental phenotype, neuroimaging, and family-based segregation context. For underrepresented settings such as Kazakhstan and Central Asia, such locally grounded clinicogenetic series are important not only for reporting variants, but also for refining phenotype interpretation, contextualizing inherited versus de novo findings, and improving counseling relevance within the population being studied [37,38].

The present study contributes a referral-based pediatric epilepsy series from Kazakhstan focused on cases with clinically relevant or potentially relevant genetic findings. The cohort includes diverse biological categories, including ion channel, mTOR pathway, mitochondrial/metabolic, synaptic and neuromuscular transmission, organelle-related, and chromatin/transcription-associated neurodevelopmental conditions. It is enriched for early-onset epilepsy, with most cases presenting in the neonatal, infantile, or early childhood period, thereby representing a clinically high-yield group for genomic evaluation. Within this heterogeneous cohort, *SCN1A* emerged as the largest recurrent gene, providing an opportunity to examine within-gene phenotypic variability alongside the wider genetic architecture of the series.

Accordingly, the aims of this study were to describe the demographic profile and age-of-onset distribution of pediatric epilepsy cases with clinically relevant or potentially relevant genetic findings in Kazakhstan; to characterize the genetic architecture of the cohort, including variant classes and inheritance/segregation patterns; and to describe the associated seizure, neurodevelopmental, electroencephalographic, neuroimaging, and syndromic phenotypes.

## 2. Materials and Methods

### 2.1. Study Design and Setting

This study was designed as a retrospective, single-center, referral-based descriptive case series of pediatric patients with epilepsy or epilepsy-associated neurodevelopmental disorders who had a genetic finding considered clinically relevant or potentially relevant to the observed phenotype. The study was conducted using clinical data collected at the “University Medical Center” Corporate Fund in Kazakhstan. The cohort was not assembled as a consecutive series of all children with early-onset or syndromic epilepsy undergoing genetic testing; therefore, the study was not designed to estimate the diagnostic yield of genetic testing in Kazakhstan or in the local referral population. Instead, the objective was to characterize the clinical, molecular, segregation, and phenotypic spectrum of genetically informative cases evaluated in a tertiary clinical setting.

The study included two temporally distinct components. First, retrospective clinical data were reviewed from patients’ first hospitalizations at the “University Medical Center” Corporate Fund between April 2023 and January 2026. Second, research-related recruitment, informed consent, sample collection, segregation analysis, and molecular genetic testing were conducted separately within the approved research framework from March 2025 to January 2026. Thus, the earlier clinical dates refer to the availability of hospitalization and phenotypic data, whereas genetic testing and other study-specific procedures were performed only after the relevant ethics approvals had been obtained.

### 2.2. Participants and Eligibility Criteria

Eligible participants were children with epilepsy or epilepsy-associated neurodevelopmental disorders who met all of the following criteria: (1) pediatric age at clinical evaluation; (2) the availability of a documented genetic result considered relevant to the phenotype; and (3) sufficient clinical information in the medical record to permit phenotypic characterization. Cases were included if seizure history, developmental information, and at least part of the electroclinical or neuroimaging dataset were available for review. Patients without adequate clinical documentation or without a genetically informative finding were excluded from the present analysis. The cohort was assembled from genetically informative referral cases rather than from a prospectively screened or consecutive testing population. A systematically defined denominator of all pediatric epilepsy patients evaluated or genetically tested during the study period was not available; therefore, diagnostic yield could not be calculated.

For the purposes of this study, a “genetically informative finding” was defined as a pathogenic or likely pathogenic variant in a gene with an established or plausible relationship to epilepsy, neurodevelopmental disease, metabolic disease, or a syndromic disorder consistent with the patient’s phenotype. VUS-only cases were included only when the variant occurred in a gene with a strong or plausible relationship to the patient’s phenotype and when additional supportive evidence was present. This evidence included one or more of the following: close phenotypic concordance with the known gene-associated disease spectrum, inheritance or segregation compatible with the suspected disease mechanism, absence of a more likely alternative molecular diagnosis, predicted functional relevance based on variant type or location, and/or supportive external evidence such as in silico predictions, gene constraint, or limited prior reports. These cases were analyzed as candidate genotype–phenotype associations and were not considered molecularly confirmed diagnoses.

### 2.3. Clinical Data Collection

Clinical data were extracted retrospectively from available medical records using a structured review framework. The following variables were collected where available: sex, age at seizure onset, seizure semiology, seizure frequency and duration, history of febrile seizures, developmental profile, speech and motor milestones, autism spectrum or behavioral features, neurological examination findings, family history, parental consanguinity status, electroencephalographic findings, neuroimaging findings, extra-neurological manifestations, anti-seizure medication exposure, and reported treatment response. Additional syndrome-relevant findings, including dysmorphic features, congenital malformations, ophthalmologic abnormalities, and hearing impairment, were also recorded when documented in the chart.

For descriptive analysis, seizure onset was categorized as neonatal (<1 month), infantile (1–12 months), or childhood onset (>12 months). Seizure semiology was grouped into broad categories including focal seizures, generalized seizures, tonic seizures, epileptic spasms/spasm-like events, and mixed or polymorphic phenotypes. Neurodevelopmental features were summarized across cognitive/developmental impairment, speech delay, motor delay, autism spectrum features, and behavioral disturbances. Where appropriate, genes were further grouped by presumed pathogenetic mechanism.

Neurodevelopmental outcomes were extracted from clinical documentation and were based on treating clinicians’ recorded assessments, caregiver-reported developmental history, and available neurologic/genetic consultation notes. Standardized developmental or cognitive testing results were not uniformly available; therefore, developmental outcomes were summarized descriptively rather than graded using formal severity scales. Developmental delay/intellectual impairment was recorded when global developmental delay, intellectual disability, or significant age-inappropriate delay was documented in the medical record. Speech delay, motor delay, autism spectrum features, behavioral disturbances, and hypotonia were recorded only when explicitly documented by the treating clinician or relevant specialist. EEG and MRI findings were abstracted from available clinical reports. Raw EEG recordings and original MRI images were not centrally re-reviewed by blinded epileptologists or neuroradiologists for this study. Therefore, EEG/MRI data were used for descriptive clinicophenotypic characterization and may reflect inter-rater and reporting variability across the original clinical evaluations.

Seizure semiology and treatment response were also extracted retrospectively from available clinical records. Because seizure frequency, duration, medication exposure, and longitudinal response were not documented with uniform detail across all cases, treatment response was summarized qualitatively as favorable, partial/transient, fluctuating, poor, or unavailable according to the wording and follow-up information available in the chart. No standardized seizure frequency diary, treatment response scale, or prospective follow-up protocol was available for the full cohort. Because follow-up duration under specific regimens, seizure frequency diaries, seizure-free intervals, and percentage seizure reductions were not consistently documented, these variables could not be summarized systematically across the cohort. Treatment observations were therefore interpreted as descriptive case-level findings rather than comparative efficacy data.

### 2.4. Sample Collection and DNA Extraction

A total of 105 genomic DNA samples were isolated from peripheral whole blood collected in 2 mL K2-EDTA tubes. These samples included DNA from the 31 pediatric probands in the analytic cohort and from available parents and siblings who participated in segregation analysis and Sanger validation. DNA extraction was performed using both automated and manual approaches. Automated extraction was carried out using the Lab-Aid 824s workstation (Zeesan Biotech Co., Ltd., Xiamen, China) in combination with the Lab-Aid 824s Blood DNA Extraction Kit. Manual extraction was performed using the PureLink™ Genomic DNA Mini Kit (Thermo Fisher Scientific, Waltham, MA, USA). All procedures were conducted in accordance with the manufacturers’ instructions.

DNA concentration and purity were evaluated using a NanoDrop™ One spectrophotometer (Thermo Fisher Scientific, Waltham, MA, USA). The mean DNA concentration was approximately 50 ng/µL. Purity ratios were consistent across both extraction methods, with A260/A280 ≈ 1.8 and A260/A230 ≈ 2.2, indicating minimal protein and organic contamination. All DNA samples were stored at −80 °C prior to downstream applications.

### 2.5. Whole-Exome Sequencing and Bioinformatics Analysis

Whole-exome sequencing (WES) was performed by a commercial service provider. Exome capture was conducted using the xGen Exome Research Panel v2, supplemented with the xGen Human mtDNA Panel and xGen Custom Hyb Panel v2 (Integrated DNA Technologies, Coralville, IA, USA). Sequencing was carried out on the NovaSeq X platform (Illumina, San Diego, CA, USA).

Sequencing reads were aligned to a GRCh38-based reference genome implemented by the commercial sequencing pipeline. This reference retained the standard GRCh38 nuclear genome sequence, with pipeline-specific masking of known falsely duplicated regions on chromosome 21 to reduce mapping artifacts, and incorporated the revised Cambridge Reference Sequence (rCRS) for mitochondrial DNA analysis. No other custom modifications to the nuclear reference sequence were applied. The mean depth of coverage across the target region was 140×, with 98.4% of bases covered at ≥20×.

Variant calling was performed using a GATK-based pipeline for single-nucleotide variants (SNVs) and small insertions/deletions (INDELs), with additional tools applied for copy number variant (CNV) detection and mitochondrial variant analysis. Variant annotation was performed using the Variant Effect Predictor (VEP, Ensembl). CNV analysis was systematically reviewed as part of the WES pipeline; however, no pathogenic or likely pathogenic CNVs relevant to the epilepsy-associated phenotypes fulfilled the inclusion criteria for this series.

Variants were prioritized and classified according to the guidelines of the American College of Medical Genetics and Genomics and the Association for Molecular Pathology (ACMG/AMP), taking into account the patient’s phenotype and relevant family history [39]. For interpretive clarity, cases were separated into two categories: (i) molecularly confirmed or strongly supported cases, defined by pathogenic or likely pathogenic variants consistent with the patient phenotype and inheritance pattern; and (ii) genetically informative but not definitively diagnostic cases, including VUS-containing cases. Heterozygous VUS in dominant epilepsy-associated genes, particularly when inherited from an apparently unaffected parent, were not considered definitive molecular diagnoses and were interpreted only as candidate or potentially contributory findings.

### 2.6. Sanger Sequencing Validation

Variants identified by WES were validated by Sanger sequencing in probands and available family members, including parents and siblings. Primers were designed using Primer3Plus and selected according to standard criteria for specificity, GC content, melting temperature, and minimal secondary structure formation. Primer sequences are listed in Supplementary Table S1. Target regions were amplified by PCR using AmpliTaq Gold™ Fast PCR Master Mix (Thermo Fisher Scientific, Waltham, MA, USA), and amplicons were verified by 1.8% agarose gel electrophoresis. PCR products were purified with the PureLink™ PCR Purification Kit (Thermo Fisher Scientific, Waltham, MA, USA) and subjected to cycle sequencing using the DNA Sanger Sequencing Kit (Suzhou Microread Genetics Co., Ltd., Suzhou, China). Sequencing products were purified by ethanol precipitation, resuspended in Hi-Di™ formamide, and analyzed on a 3500 Genetic Analyzer (Thermo Fisher Scientific, Waltham, MA, USA) according to the manufacturers’ protocols. Sequence traces were reviewed using Vector NTI software version 11.5.3.

### 2.7. Data Analysis

The study was primarily descriptive. Categorical variables were summarized as counts and percentages, while continuous variables such as age at seizure onset were summarized using median and range or range alone, depending on the distribution and completeness of available data. Because of the modest cohort size, marked genetic heterogeneity, and variable completeness of retrospective documentation, no formal comparative hypothesis testing was planned for the full cohort. Denominators were reported explicitly for variables with missing data. Case-level synthesis was used for recurrent genes and clinically important subgroups, particularly *SCN1A*, to explore genotype–phenotype patterns. All analyses were performed at the descriptive level. For the recurrent *SCN1A* subgroup, phenotypic classification was performed descriptively using features emphasized in the contemporary ILAE and Dravet/GEFS+ literature, including age at seizure onset, febrile or temperature sensitivity, seizure semiology and multiplicity, developmental course, EEG findings, and overall clinical severity [40,41,42,43].

### 2.8. Ethics

This study was approved by the Local Ethics Committee of the National Laboratory Astana (No. 03-2024, 2 October 2024) and the Local Ethics Committee of the Corporate Fund “University Medical Center” (No. 3/2025/ПЭ, 28 April 2025). The retrospective clinical review included data from first hospitalizations between April 2023 and January 2026, whereas research-related recruitment, informed consent, sample collection, and genetic analyses were conducted from March 2025 to January 2026 under the approved study framework. The study was conducted in accordance with institutional ethical standards and the principles of the Declaration of Helsinki. Written informed consent was obtained from the legal guardians of all minor participants prior to inclusion in the study, in accordance with the principles of the Declaration of Helsinki. Additional written informed consent was also obtained from participating family members for trio-based segregation analysis and Sanger validation, where applicable. Written informed consent for publication of de-identified clinical and genetic data was obtained from participants’ legal representatives.

## 3. Results

### 3.1. Demographic and Age-of-Onset Characteristics

A total of 31 pediatric patients with epilepsy or epilepsy-associated neurodevelopmental disorders and clinically relevant or potentially relevant genetic findings were included in the cohort (Table 1, Supplementary Table S1), including 15 males (48.4%) and 16 females (51.6%). Seizure onset occurred predominantly in early life, with a median onset age of 5.0 months (IQR, 2.0–13.0 months). Neonatal-onset epilepsy was observed in 4/31 patients (12.9%), infantile onset in 18/31 (58.1%), and childhood onset in 9/31 (29.0%), indicating that more than two-thirds of cases presented within the first year of life.

Based on retrospective clinical documentation, developmental delay and/or intellectual impairment was recorded in 20 patients (64.5%), while speech delay and motor delay were documented in 17 (54.8%) and 15 (48.4%), respectively. Autism spectrum features and behavioral disturbances were less frequent, each recorded in 3 patients (9.7%).

Among neurologic examination findings, hypotonia was identified in 12 patients (38.7%), whereas ataxia was less common and observed in 2 cases (6.5%). Syndromic and extra-neurological manifestations were also notable: dysmorphic features were present in 11 patients (35.5%), congenital malformations in 6 (19.4%), and vision abnormalities in 9 (29.0%). Hearing abnormalities were uncommon, occurring in only 1 patient (3.2%).

### 3.2. Molecular Findings and Segregation Patterns

The most recurrent gene was *SCN1A*, identified in 8/31 patients (25.8%), making *SCN1A*-related epilepsy the largest single molecular subgroup in the cohort (Table 2). Other recurrent genes included *TSC1* and *TSC2*, each represented by two cases, whereas the remaining genes were observed in single patients. Most cases carried pathogenic or likely pathogenic variants consistent with the clinical phenotype and were therefore interpreted as molecularly confirmed or strongly supported diagnoses. However, 5/31 patients (16.1%) had VUS-only findings, including heterozygous variants in dominant epilepsy-associated genes inherited from apparently unaffected parents. These cases included candidate findings in *TSC1*, *DEPDC5*, *SCN2A*, *SCAF4*, and *KCNQ5*. They were retained because the genes were considered clinically plausible in relation to the presenting phenotype and because no more likely alternative molecular diagnosis was identified, but they were not interpreted as definitive molecular diagnoses. In particular, VUS-only findings were considered candidate or potentially contributory variants requiring additional segregation, functional, population-frequency, or case-level evidence for reclassification. Therefore, all VUS-related genotype–phenotype observations should be interpreted as descriptive and hypothesis-generating. No clinically relevant pathogenic or likely pathogenic CNVs were detected in the analytic cohort.

Segregation analysis demonstrated that de novo occurrence was the most frequent pattern overall, particularly among dominant channelopathy and developmental encephalopathy genes. Confirmed or presumed de novo variants were observed in multiple cases involving *SCN1A*, *KCNQ2*, *ATP7A*, *CDKL5*, *SMC1A*, *TSC1*, *PIGA*, and *SLC6A8*. In contrast, biallelic inheritance was identified in several metabolic and organelle disorders, including *NDUFV1*, *ECHS1*, *PEX1*, and *PNPO*, consistent with autosomal recessive disease mechanisms. Maternal or paternal inheritance with an apparently unaffected parent was noted in selected cases, including *SCN2A*, *SCN8A*, *DEPDC5*, *KCNQ5*, *TSC1*, and *SCAF4*. In several cases, parental testing was incomplete or unavailable, limiting definitive segregation interpretation.

Taken together, these findings illustrate the broad spectrum of clinically relevant or potentially relevant genetic findings in this pediatric epilepsy cohort, with a predominance of early-onset channelopathies and a substantial contribution from metabolic, mTOR-related, and neurodevelopmental disorders.

Family history was informative in several cases. In the *MECP2* case (Case 6), multiple relatives reportedly had severe childhood neurodevelopmental phenotypes, although these were not genetically resolved and could not be directly linked to the identified variant. The *PRRT2* case (Case 10) showed the clearest familial support, with an affected sibling reportedly carrying the same variant. In the *DEPDC5* case (Case 18), a sibling had severe infantile-onset refractory epilepsy with a fatal outcome, but interpretation remained limited because the detected variant was a maternally inherited VUS. Familial aggregation was also observed in the *TSC1* case (Case 23), with paternal seizures, and in the *TSC2* case (Case 28), which showed a strong multigenerational history of TSC-associated manifestations and epilepsy.

### 3.3. Clinical Phenotype Spectrum

Most patients presented with early-onset epilepsy, with seizure onset within the first year of life in 22/31 cases (71.0%). Seizure semiology was heterogeneous. Focal seizure phenotypes were most frequent and included focal clonic seizures in *KCNQ2* (Case 4) and *PEX1* (Case 12), focal motor seizures with versive or myoclonic features in several *SCN1A* cases (Cases 3, 14, 24), focal seizures with tonic and autonomic/oral automatisms in *SMC1A* (Case 21), focal autonomic-tonic seizures in *DEPDC5* (Case 18), and focal-to-bilateral tonic–clonic evolution in *SCN1A* and *TSC2* cases (Cases 22, 28, 29). Generalized tonic–clonic or generalized seizure phenotypes were observed in *SLC6A8* (Case 1), *SCN1A* (Cases 2, 16), *NDUFV1* (Case 9), *IBA57* (Case 11), and *MECP2* (Case 6). Tonic seizures were seen in *TSC1* (Case 8), *ECHS1* (Case 15), *SCN2A* (Case 25), and *PIGA* (Case 27), whereas epileptic spasms or spasm-like events were documented in *ATP7A* (Case 5), *SCAF4* (Case 26), *TSC2* (Case 29), *KCNQ5* (Case 30), and electroclinically in *NDUFV1* (Case 9). A clustered myoclonic/startle-like, febrile-sensitive phenotype was noted in *PNPO* deficiency (Case 31).

Neurodevelopmental outcomes ranged from preserved development to profound impairment. Development was described as age-appropriate or broadly preserved in *SCN1A* (Cases 2, 16, 22, 24), *PRRT2* (Case 10), *DEPDC5* (Case 18), *SCN2A* (Case 25), and *SLC5A7* (Case 7). In contrast, severe or profound developmental impairment—with absent or markedly limited speech and major motor delay—was observed in *SCN1A* (Case 3), *ATP7A* (Case 5), *NDUFV1* (Case 9), *IBA57* (Case 11), *PEX1* (Case 12), *ECHS1* (Case 15), *SMC1A* (Case 21), and *PIGA* (Case 27). Developmental regression was documented in the *MECP2* case (Case 6). Autism spectrum features were recorded in *SLC6A8* (Case 1), *SCN1A* (Case 14), and *SCN8A* (Case 19).

EEG abnormalities were present in most evaluated patients and were frequently sleep-activated. This pattern was seen in *MECP2* (Case 6), *SLC5A7* (Case 7), *CDKL5* (Case 13), several *SCN1A* cases (Cases 14, 20, 22, 23), *SCN2A* (Case 25), *SCAF4* (Case 26), *TSC2* (Case 28), and *KCNQ5* (Case 30). More severe encephalopathic phenotypes, including *ATP7A*, *PEX1*, *ECHS1*, *PNPO*, and *NDUFV1* (Cases 5, 12, 15, 31, and 9), showed markedly abnormal EEGs with multifocal or high-index epileptiform activity and/or electroclinical seizures. A photoparoxysmal response was reported in one *SCN1A* case (Case 22). By contrast, routine EEG was non-diagnostic or largely normal in some milder cases, including *SCN1A* (Case 16) and *DEPDC5* (Case 18).

Neuroimaging findings ranged from nonspecific white matter changes to syndrome-associated patterns. Imaging compatible with tuberous sclerosis complex was present in *TSC1*/*TSC2* cases (Cases 8, 23, 28, 29). A Leigh-like or mitochondrial/metabolic pattern was seen in *NDUFV1*, *ECHS1*, and *PIGA* (Cases 9, 15, 27), while IBA57 showed a leukodystrophy-like pattern with callosal involvement (Case 11). *PEX1* was associated with agenesis of the corpus callosum and colpocephaly (Case 12), and *SCN1A* Case 3 showed occipito-parietal hypomyelination. Hippocampal abnormalities were noted in *CDKL5* (Case 13) and in *SCN1A*/*KCNQ5* cases with suspected malrotation (Cases 16, 30). Several cases, including *SCN1A* (Cases 2, 17, 22), *KCNQ2* (Case 4), *PRRT2* (Case 10), and *SCN2A* (Case 25), had no major structural lesion or only mild nonspecific abnormalities.

Treatment response was variable and was summarized qualitatively because follow-up duration under specific regimens, seizure frequency diaries, seizure-free intervals, and percentage seizure reductions were not consistently available across cases (Table 3; Supplementary Table S1). More favorable responses were reported in *PRRT2* (Case 10; carbamazepine), *PNPO* (Case 31; initial pyridoxine response), and one *SCN1A* case after stiripentol introduction (Case 14). Partial, transient, or fluctuating improvement was reported in *SLC6A8*, *TSC1*, *PEX1*, *SCN1A*, and *SCN2A* cases (Cases 1, 8, 12, 20, 22, 23, 25), whereas persistent seizures despite multiple anti-seizure medications (ASM) were common in *SCN1A* (Cases 3, 17, 24), *KCNQ2* (Case 4), *ATP7A* (Case 5), *CDKL5* (Case 13), *DEPDC5* (Case 18), *SCAF4* (Case 26), *TSC2* (Case 28), and *KCNQ5* (Case 30). Vagus nerve stimulation was performed in one severe *SCN1A* case (Case 3).

Extra-neurological manifestations were frequent. Hypotonia was documented across multiple disorders, including *SLC6A8*, *KCNQ2*, *ATP7A*, *TSC1*, *NDUFV1*, *PEX1*, *ECHS1*, *PIGA*, and *PNPO* (Cases 1, 4, 5, 8, 9, 12, 15, 27, 31). Dysmorphic features were noted in *ATP7A*, *PEX1*, *SMC1A*, *SCN1A*, *SCAF4*, *PIGA*, and *PNPO* (Cases 5, 12, 21, 24, 26, 27, 31). Cutaneous findings were prominent in *TSC1*/*TSC2* cases and were also reported in *SCN1A* (Case 2) and *PNPO* (Case 31). Cardiac anomalies were present in *SCN1A*, *KCNQ2*, and *PIGA* (Cases 2, 4, 27), while visual abnormalities occurred in *SCN1A*, *NDUFV1*, *TSC1*/*TSC2*, *SCAF4*, and *PIGA*-related disease. Additional syndrome-associated features included abnormal hair in *ATP7A* (Case 5), a congenital myasthenic syndrome-like phenotype in *SLC5A7* (Case 7), Rett-like regression and stereotypies in *MECP2* (Case 6), and CdLS-like dysmorphism in *SMC1A* (Case 21).

### 3.4. SCN1A-Related Phenotypic Spectrum

Because *SCN1A* represented the largest recurrent gene in the cohort, cases with SCN1A variants were examined separately using a descriptive phenotypic continuum framework based on age at onset, seizure characteristics, developmental course, EEG findings, and overall clinical severity (Table 4). Overall, the subgroup showed a broad phenotypic continuum, ranging from early-onset Dravet-compatible epilepsies to later-onset, developmentally milder non-Dravet phenotypes.

Two cases were classified as Dravet syndrome/Dravet-compatible. Case 2 had seizure onset at 6 months, a de novo nonsense variant, generalized tonic–clonic seizures, and sleep interictal epileptiform discharges, consistent with an early *SCN1A*-related epileptic encephalopathy. Case 3, with onset at 2 months and a de novo missense variant, showed a more severe course characterized by focal motor seizures with myoclonic features, marked developmental impairment, drug-resistant epilepsy, and subsequent vagus nerve stimulation, supporting placement within the Dravet-spectrum.

Two additional cases were classified as Dravet-spectrum/borderline Dravet. Case 17 presented at 4 months with focal seizures and persistent seizures despite multiple anti-seizure medications, but developmental involvement was less pronounced than in the most severe case. Case 20, with onset at 6 months, had focal non-motor seizures with tonic–clonic evolution, mild developmental delay, persistent eyelid myoclonia, and multifocal/bilateral sleep-activated epileptiform abnormalities, also supporting an intermediate position close to the Dravet end of the spectrum.

One case (Case 14) was classified as intermediate *SCN1A*-related epilepsy. This patient presented at 9 months with focal motor seizures with versive and clonic features, speech delay, autism spectrum features, and preserved gross motor development. The phenotype was more developmentally affected than typical genetic epilepsy with febrile seizures plus (GEFS+), but less severe than classical Dravet syndrome. Improved seizure control after the addition of stiripentol further supported its clinical relevance within the *SCN1A* spectrum.

Three cases were categorized as GEFS+/non-Dravet *SCN1A*-related epilepsy. Case 16 had late seizure onset at 47 months, generalized tonic–clonic seizures, and broadly preserved development. Case 22 presented at 11 months with focal-to-bilateral tonic–clonic seizures, preserved development, and a comparatively milder course despite abnormal EEG findings. Case 24 had the latest onset in the subgroup (72 months), focal motor seizures, preserved development, and focal ictal abnormalities on EEG/PET, supporting classification within the non-Dravet *SCN1A* spectrum.

Taken together, the *SCN1A* subgroup in this cohort supports a continuum model rather than a binary separation between Dravet syndrome and GEFS+. Earlier seizure onset, developmental involvement, and incomplete response to treatment tended to cluster toward the Dravet-compatible end, whereas later onset and preserved development were more typical of the non-Dravet group.

## 4. Discussion

This referral-based case series highlights the marked clinical and molecular heterogeneity of pediatric epilepsy cases with clinically relevant or potentially relevant genetic findings in Kazakhstan. The cohort was enriched for early-onset disease, with most patients presenting in the first year of life, and many showed developmental impairment, EEG and MRI abnormalities, and syndromic extra-neurological features. Ion channel genes, particularly *SCN1A*, represented the largest molecular subgroup, while mTOR-related, mitochondrial/*metabolic*, organelle-related, synaptic, and neurodevelopmental/chromatin-associated genes were also represented. Together, these findings support the view that clinically selected pediatric epilepsy cases referred for genomic evaluation are best understood as a broad clinicogenetic spectrum rather than a single diagnostic entity.

### 4.1. Molecular Architecture

The molecular findings in this cohort were dominated by ion channel genes (12/31, 38.7%), followed by mitochondrial/metabolic genes (6/31, 19.4%), mTOR pathway genes (5/31, 16.1%), and neurodevelopmental/chromatin/transcriptional genes (4/31, 12.9%), with smaller contributions from synaptic/neuromuscular transmission and organelle-related genes. This distribution is biologically plausible and consistent with contemporary epilepsy genomics literature, in which channelopathies remain major contributors to early-life epilepsies and developmental/epileptic encephalopathies, while mTOR pathway, metabolic, and transcription/chromatin-associated genes account for clinically important syndromic and neurodevelopmentally complicated presentations. mTOR pathway disorders are particularly relevant because they connect epilepsy with structural brain abnormalities and broader neurodevelopmental impairment, whereas mitochondrial and metabolic epilepsies often present with multisystem disease and characteristic neuroimaging signatures [17,18,61].

In relation to international pediatric epilepsy sequencing cohorts, the overall pattern of this series is broadly consistent with the enrichment of ion channel, synaptic, metabolic, mTOR pathway, and transcriptional/chromatin-related genes among children with early-onset, syndromic, or developmental and epileptic encephalopathy phenotypes. However, direct comparison of gene frequencies should be made cautiously because international cohorts differ substantially in inclusion criteria, testing strategy, age at onset, prior negative testing, parental testing availability, and whether VUS are reported. Unlike population-based or consecutive diagnostic cohorts, the present series was assembled from genetically informative referral cases and therefore cannot be used to compare diagnostic yield or true gene prevalence with international studies.

The recurrence of *SCN1A* in 8/31 patients (25.8%) is consistent with the prominent role of *SCN1A* in early-onset and developmental epilepsy cohorts, but appears higher than the proportion reported in several larger international sequencing series. For example, in a large East Asian pediatric epilepsy cohort from Korea, *SCN1A* was the most frequently implicated gene, accounting for 49/957 tested patients (5.1%) and 49/310 genetically diagnosed patients (15.8%) [62]. In an infantile-onset pharmacoresistant epilepsy cohort undergoing exome sequencing as a first-tier test, *SCN1A* was also the most common disease-causing gene, identified in 13/103 patients (13.0%) [63]. A national Italian survey of genetic developmental and epileptic encephalopathies similarly reported *SCN1A* as the most frequent single-gene etiology, accounting for approximately 16% of cases [64]. In contrast, some European early-infantile developmental and epileptic encephalopathy cohorts have reported other genes, such as *KCNQ2*, *MECP2*, *FOXG1*, *IQSEC2*, *KMT2A*, and STXBP1, among the leading contributors, illustrating the strong influence of cohort definition, age at onset, syndrome composition, and testing strategy on apparent gene frequencies [13]. Therefore, the relatively high proportion of *SCN1A* findings in the present series should not be interpreted as evidence of a Kazakhstan-specific genetic signature. Rather, it most likely reflects the referral-based enrichment of early-onset, Dravet-spectrum, febrile-sensitive, and developmentally complicated epilepsy cases, together with the fact that the cohort was restricted to genetically informative cases rather than all tested children.

These findings should, however, be interpreted in light of study design. The present series is not a population-based epilepsy sample, but a high-yield referral cohort composed of clinically selected children with suspected genetic disease and enriched for early-onset presentations. Such ascertainment is expected to increase the representation of severe, syndromic, developmental, and genetically identifiable epilepsies relative to unselected epilepsy populations, because tertiary referral pathways preferentially capture children with refractory seizures, developmental impairment, multisystem features, abnormal EEG/MRI findings, or complex diagnostic histories. In this context, the prominence of *SCN1A* findings, mTOR pathway findings, and mitochondrial/metabolic findings likely reflects both true biological burden and referral bias toward more complex pediatric cases [17,65,66].

Another noteworthy feature of the cohort is the proportion of variants that, within the present curation, appeared to be previously unreported or incompletely cataloged. Among 36 reportable variants listed in Table 2, 18 (50.0%) had no cited peer-reviewed precedent, and 13 (36.1%) had neither cited literature support nor ClinVar evidence. At the patient level, 17 of 31 cases (54.8%) included at least one variant without cited prior peer-reviewed evidence, and 12 of 31 (38.7%) included at least one variant lacking both cited literature and ClinVar support [65,67]. However, absence from the available literature or ClinVar does not establish global novelty or pathogenicity, particularly in the absence of functional validation, complete segregation, or independent recurrence. These variants should therefore be regarded as putatively unreported, candidate, or incompletely cataloged observations that require additional evidence for confirmation and possible reclassification. Importantly, pathogenic/likely pathogenic variants and VUS-based observations were interpreted separately: the former were considered molecularly confirmed or strongly supported findings, whereas VUS-only cases were reported to illustrate candidate genotype–phenotype associations and cannot be considered definitively causal at this stage.

The inheritance structure of the cohort further supports the clinical relevance of segregation-informed interpretation. As expected in a severe pediatric referral series, de novo variants were frequent among dominant channelopathy and developmental encephalopathy genes, especially *SCN1A*, and were also observed in *KCNQ2*, *ATP7A*, *SLC6A8*, *CDKL5*, *SMC1A*, *TSC1*, and *PIGA*. In contrast, biallelic inherited variants clustered in mitochondrial/metabolic and organelle-related disorders such as *NDUFV1*, *ECHS1*, *PEX1*, and *PNPO*. Several dominant or X-linked cases showed parental transmission from apparently unaffected or only mildly affected parents, underscoring the importance of cautious interpretation. This issue is illustrated by Case 25, in which an *SCN2A* VUS was inherited from an apparently unaffected father, and Case 19, in which an *SCN8A* likely pathogenic variant was inherited from an apparently unaffected mother. Similar caution applies to heterozygous VUS in dominant epilepsy-associated genes such as *DEPDC5*, *KCNQ5*, and *SCAF4*. Such patterns may reflect incomplete penetrance, variable expressivity, undetected parental mosaicism, or incomplete parental phenotyping; however, they also weaken causal inference and require additional segregation, functional, population-frequency, and independent case-level evidence before causal interpretation is justified. Accordingly, these inherited or VUS-based findings were not treated as definitive molecular diagnoses. This pattern is consistent with the current diagnostic practice literature emphasizing the value of trio-based and segregation-informed interpretation in pediatric epilepsy, particularly for confirming de novo events, clarifying recessive inheritance, and contextualizing incompletely penetrant, variably expressive, or uncertain inherited variants [58,59]. In our cohort, family-based data were especially informative in selected cases, including *PRRT2* and *TSC2*, where segregation or family history supported clinical interpretation, as well as candidate findings such as *DEPDC5* and selected *TSC1* variants, where segregation context highlighted the need for cautious interpretation rather than confirming pathogenicity.

### 4.2. Clinical Phenotype Burden and the SCN1A-Related Phenotypic Continuum

The clinical burden in this cohort extended well beyond seizures alone. Developmental delay/intellectual impairment (20/31, 64.5%), speech delay (17/31, 54.8%), motor delay (15/31, 48.4%), hypotonia (12/31, 38.7%), dysmorphic features (11/31, 35.5%), vision abnormalities (9/31, 29.0%), and congenital malformations (6/31, 19.4%) were all common, supporting the view that many clinically selected pediatric epilepsy cases with relevant genetic findings are better understood within a broader neurodevelopmental or multisystem framework rather than as isolated seizure syndromes. This pattern is consistent with recent studies of developmental and epileptic encephalopathies and pediatric epilepsy genomics, which emphasize that epilepsy commonly co-occurs with cognitive, speech, motor, behavioral, and systemic manifestations, and that these associated features often provide important etiologic clues [67,68]. However, these neurodevelopmental categories were based on retrospective clinical documentation rather than standardized developmental testing, and severity or timing of assessment could not be harmonized across cases.

EEG abnormalities were common and frequently sleep-activated, but the observed patterns were heterogeneous, ranging from focal epileptiform discharges to multifocal or high-index abnormalities and electroclinical seizures in the most severe encephalopathic presentations. MRI findings were similarly diverse, including tuberous sclerosis complex-compatible lesions, Leigh-like or metabolic-pattern deep gray/brainstem abnormalities, callosal anomalies, leukodystrophy-like white matter involvement, and hippocampal sclerosis or malrotation. These observations align with published data showing that confirmed genetic epilepsies can have mechanism-specific neurophysiologic and imaging signatures: TSC-related epilepsies commonly combine structural cortical/subependymal lesions with epileptogenic network abnormalities, whereas mitochondrial and Leigh-spectrum epilepsies often show characteristic bilateral basal ganglia, brainstem, cerebellar, or diffuse metabolic-pattern changes [69,70,71]. Accordingly, in suspected or confirmed genetically mediated pediatric epilepsy, EEG and MRI should be viewed not only as tools for seizure localization, but also as components of broader syndrome definition.

The *SCN1A* subgroup deserves separate consideration because *SCN1A* was the largest recurrent gene in the cohort and illustrated a clinically meaningful continuum rather than a strict binary distinction between Dravet syndrome and GEFS+. Cases with *SCN1A* variants ranged from early-onset Dravet-compatible and borderline Dravet presentations to later-onset, developmentally milder non-Dravet phenotypes, with age at onset, developmental trajectory, and treatment response serving as the most practical stratifiers. This interpretation is concordant with modern *SCN1A* literature, which recognizes that pathogenic or likely pathogenic *SCN1A* variants can be associated with phenotypes spanning febrile seizure-plus and GEFS+-range phenotypes through intermediate developmental epilepsies to classic Dravet syndrome, with overlap rather than absolute categorical boundaries [41,42,43]. In our cohort, earlier-onset and more developmentally affected cases clustered toward the Dravet-compatible end, whereas later-onset cases with preserved development fit better within the non-Dravet spectrum; however, this subgrouping should be understood as descriptive rather than as a definitive nosologic reassignment.

### 4.3. Treatment Implications and Precision-Medicine Relevance

Treatment response in this cohort was more often partial, fluctuating, or poor than clearly favorable, particularly across recurrent *SCN1A* cases, *TSC1*/*TSC2*-variant cases, and several severe metabolic or developmental encephalopathy presentations. This is consistent with the broader literature showing that many monogenic pediatric epilepsies, especially developmental and epileptic encephalopathies, remain difficult to control despite sequential or combined antiseizure therapy. In this context, the treatment data from our series are clinically important not because they show uniformly targetable disease, but because they highlight a small number of genotype-linked therapeutic signals within an otherwise pharmacoresistant landscape. The clearest favorable responses were observed in the *PRRT2* case treated with carbamazepine, the *PNPO* case with an initial response to pyridoxine, and one *SCN1A* case with improved seizure control after stiripentol, whereas most other genotype groups showed only partial or poor control. The *PRRT2* observation is concordant with cohort data showing that pathogenic *PRRT2*-associated self-limited infantile epilepsy often responds well to sodium channel blockers, including carbamazepine, with higher response rates than several alternative agents [72,73]. Similarly, the *PNPO* finding is clinically plausible because *PNPO* deficiency belongs to the spectrum of vitamin B6-responsive epilepsies, although published series also emphasize that responsiveness may vary between pyridoxine and pyridoxal-5′-phosphate and that some patients show only partial or context-dependent benefit [74].

The *SCN1A* subgroup further illustrates why molecular diagnosis, when supported by pathogenic or likely pathogenic variants and phenotype concordance, can have value beyond etiologic labeling alone. In one *SCN1A*-positive patient, seizure control improved after the introduction of stiripentol, which is consistent with real-world series supporting stiripentol as an effective adjunctive therapy in Dravet syndrome, particularly in *SCN1A*-positive patients [75,76]. Conversely, Case 22, who carried a pathogenic nonsense/truncating *SCN1A* variant (p.Arg1636*), showed seizure worsening on lamotrigine. This is clinically relevant because lamotrigine and related sodium channel-blocking drugs have long been recognized as capable of exacerbating seizures in many patients with *SCN1A*-related Dravet-spectrum epilepsy, particularly when loss-of-function mechanisms are suspected, even though occasional exceptions have been reported [76,77,78]. Taken together, these case-level observations indicate that well-supported molecular findings in pediatric epilepsy may do more than assign an etiologic label: it can also refine medication choice, identify potentially beneficial syndrome-specific therapies, and warn against treatments that may worsen seizure control in susceptible genotypes.

### 4.4. Relevance to Kazakhstan and Underrepresented Populations

The present study should be interpreted in the context of both the evolving epilepsy research landscape in Kazakhstan and the broader underrepresentation of many populations in clinical genomics. Although epilepsy has been increasingly characterized at the national level in Kazakhstan through epidemiologic, health system, and service delivery studies, detailed pediatric clinicogenetic datasets remain comparatively limited. Existing Kazakhstani genomic epilepsy publications have included a priority-setting paper on infantile and childhood epileptic encephalopathies and early sequencing-based studies of children with early-onset epilepsy, both of which highlighted the need for deeper molecular and phenotypic characterization in the local setting [34,35,36]. Against this background, the current series adds a clinically detailed cohort in which molecular findings are interpreted together with seizure semiology, developmental profile, EEG, MRI, and family-based segregation data, rather than as variant reports alone. This is particularly relevant because underrepresentation in genomics can constrain variant interpretation, increase uncertainty in pathogenicity assessment, and limit the transferability of precision-medicine frameworks to populations that are not well represented in reference datasets [25,26,27,37,38].

From a local clinical–practice perspective, these findings support several pragmatic priorities. First, infants and children with early-onset seizures accompanied by developmental delay, abnormal neurologic examination, dysmorphic or extra-neurological features, or unexplained EEG/MRI abnormalities should be considered high-priority candidates for genetic evaluation. Second, trio-based testing and family segregation analysis should be prioritized whenever feasible, because these data are critical for confirming de novo variants, resolving recessive inheritance, and avoiding overinterpretation of inherited VUS. Third, variant interpretation should be embedded within multidisciplinary review involving pediatric neurology, clinical genetics, laboratory genetics, neuroradiology, and genetic counseling, especially for VUS-containing cases. Finally, selected molecular findings may inform clinical management, including syndrome-specific surveillance, recurrence risk counseling, cascade testing, and, in limited but important contexts, genotype-informed medication decisions such as *PRRT2*-associated carbamazepine responsiveness, *PNPO*-related vitamin B6 therapy, or avoidance of potentially aggravating sodium channel blockers in *SCN1A*-related Dravet-spectrum presentations.

Locally generated clinicogenetic data are important not only for documenting molecular findings, but also for improving counseling relevance, clarifying inheritance and segregation patterns, and anchoring variant interpretation in a population-specific clinical context. A concrete example of this added value is provided by the IBA57 family represented in our cohort (Case 11). In the present series, this patient carried compound heterozygous *IBA57* variants, c.286T>C (p.Tyr96His) and c.667C>G (p.Arg223Gly), with biparental inheritance. Notably, this family has already been described in a separate Kazakhstani case report focused on prenatal genetic testing, in which the same variants were confirmed by Sanger sequencing in the proband and parents and were used to support reproductive counseling and prenatal risk assessment [79]. The current study complements that report by situating the family within a broader clinicogenetic pediatric epilepsy cohort and emphasizing the associated seizure, developmental, electroencephalographic, and neuroimaging phenotype. More broadly, previous Kazakhstani reports have already demonstrated the feasibility of sequencing-based evaluation in epilepsy and identified novel or rarely reported variants and phenotype variability; the current study does not replace those earlier contributions, but extends them by providing a broader clinicophenotypic synthesis and a more explicit analysis of inheritance, segregation, and recurrent subgroup patterns such as *SCN1A*. In this sense, the study contributes incrementally, but meaningfully, to the emerging evidence base for regional pediatric epilepsy genomics in Kazakhstan and Central Asia [34,35,36].

### 4.5. Strengths and Limitations

This study has several strengths. First, it provides a genetically characterized pediatric epilepsy cohort from Kazakhstan, a setting in which clinicogenetic data remain limited. Second, it integrates molecular findings with detailed clinical phenotyping, including seizure semiology, developmental profile, EEG, MRI, extra-neurological manifestations, and treatment history, allowing for interpretation beyond variant detection alone. Third, the inclusion of inheritance and segregation information, including trio- or family-based validation where available, strengthens variant interpretation and improves the clinical relevance of the findings. Fourth, the cohort captures a broad molecular spectrum, spanning ion channelopathies, mTOR-related disorders, mitochondrial/metabolic disease, organelle-related disorders, synaptic disorders, and neurodevelopmental/chromatin-associated conditions. Finally, the focused analysis of the *SCN1A* subgroup provides additional insight into within-gene phenotypic heterogeneity and supports a spectrum-based interpretation of *SCN1A*-related epilepsy.

Several limitations should also be acknowledged. First, several limitations arise from the study design. This study was based on a retrospective, single-center, referral-based case series and therefore does not represent the population-level molecular epidemiology of pediatric epilepsy in Kazakhstan. Because patients were included after identification of a clinically relevant or potentially relevant genetic finding, the cohort is subject to substantial selection and referral bias. Accordingly, this study cannot estimate the diagnostic yield of whole-exome sequencing (WES) or other genetic testing among children with early-onset or syndromic epilepsy in Kazakhstan. Calculation of diagnostic yield would require a consecutive or systematically ascertained testing cohort with a clearly defined denominator, including both positive and negative genetic test results. The cohort is likely enriched for early-onset, severe, syndromic, and genetically suspected cases, which may overrepresent monogenic and developmentally complicated epilepsies. Because the study was conducted at a tertiary referral center, children with refractory seizures, developmental delay, dysmorphic or extra-neurological features, abnormal EEG/MRI findings, or prior suspicion of a genetic syndrome were more likely to be referred and genetically evaluated than children with milder or self-limited epilepsy. Therefore, the relative contribution of mechanistic categories, such as channelopathies, mTOR pathway disorders, and mitochondrial/metabolic disorders, should not be extrapolated to unselected national pediatric epilepsy cohorts.

Second, several limitations reflect the retrospective nature of clinical data collection and broader structural constraints affecting genomic epilepsy research in this setting. The sample size was modest, and marked genetic and phenotypic heterogeneity limited formal comparative analyses across subgroups. Clinical data completeness varied across cases, particularly for seizure frequency, treatment history, longitudinal developmental follow-up, standardized neurodevelopmental assessment, and family testing. Neurodevelopmental outcomes were based on retrospective chart documentation rather than uniform cognitive or developmental scales, and severity grading or timing of assessment could not be standardized. Treatment response data were qualitative and incomplete for some patients, limiting interpretation of genotype-linked therapeutic observations. In addition, neurophysiologic and neuroimaging data were derived from available clinical reports rather than centralized blinded EEG or MRI re-review, introducing potential inter-rater and reporting bias. These limitations should be distinguished from broader system-level challenges, including restricted access to systematic trio-based testing, variable availability of longitudinal phenotyping, and limited infrastructure for centralized multidisciplinary re-review.

Finally, several limitations relate to variant interpretation. The inclusion of selected VUS-containing cases increases the descriptive value of the cohort but limits the certainty of some genotype–phenotype interpretations. This limitation is particularly relevant for heterozygous VUS in dominant epilepsy-associated genes inherited from apparently unaffected parents, where current evidence is insufficient to assign pathogenicity or to treat the variant as a definitive molecular diagnosis. Variants described as putatively unreported or incompletely cataloged should also be interpreted cautiously, because database absence does not establish global novelty or pathogenicity. Functional validation, complete segregation analysis, population frequency assessment in ancestry-relevant datasets, and independent case recurrence would be required to support reclassification or stronger claims regarding allelic expansion.

## 5. Conclusions

In conclusion, this study expands the clinicogenetic characterization of pediatric epilepsy cases with clinically relevant or potentially relevant genetic findings in Kazakhstan by describing a referral-based cohort enriched for early-onset, developmentally complicated, and syndromic epilepsy. The cohort showed marked molecular heterogeneity, with recurrent representation of ion channel disorders, particularly *SCN1A*, alongside mTOR-related, mitochondrial/metabolic, organelle-related, synaptic, and neurodevelopmental/chromatin disorders. The findings further demonstrate that these clinically selected pediatric epilepsy cases are commonly associated with substantial developmental, electroclinical, neuroimaging, and extra-neurological burden, supporting a syndromic rather than seizure-only framework for clinical evaluation. The *SCN1A* subgroup illustrated a clinically relevant phenotypic continuum from Dravet-compatible to non-Dravet presentations, while treatment observations highlighted the practical value of molecular diagnosis beyond etiologic labeling alone in selected genotype-informed contexts. Together, these data contribute to the emerging regional evidence base for pediatric epilepsy genomics in Kazakhstan and underscore the need for larger multicenter studies, broader trio-based testing, consecutive genetic testing cohorts with defined denominators, and harmonized longitudinal phenotyping in underrepresented populations.

## Figures and Tables

**Table 1:** Demographic, developmental, and clinical characteristics of cohort (*N* = 31).

Characteristics	*n*	%
**Sex**		
Male	15	48.4
Female	16	51.6
**Epilepsy age of onset**		
Neonatal onset (<1 month)	4	12.9
Infantile onset (1–12 months)	18	59.1
Childhood onset (>12 months)	9	29.0
**Neurodevelopmental features**		
Developmental delay/intellectual impairment	20	64.5
Speech delay	17	54.8
Motor delay	15	48.4
Autism spectrum disorder (ASD)	3	9.7
Behavioral disturbances	3	9.7
**Neurologic examination findings**		
Hypotonia	12	38.7
Ataxia	2	6.5
**Syndromic/extra-neurological features**		
Dysmorphic features	11	35.5
Congenital malformations	6	19.4
Vision abnormalities	9	29.0
Hearing abnormalities	1	3.2

Neurodevelopmental, neurologic examination, and syndromic/extra-neurological features are not mutually exclusive; individual patients could contribute to more than one category.

**Table 2:** Clinically relevant or potentially relevant genetic findings, ACMG classification, and inheritance/segregation patterns.

Case ID (Sex)	Gene	Variant	Protein Effect	ACMG Class	ClinVar Evidence	Inheritance/Segregation	Reported Evidence, Ref.
**Ion channelopathies**							
Case 2 (M)	*SCN1A*	c.4547C>A	p.Ser1516*	P	P	AD; de novo	[44]
Case 3 (M)	*SCN1A*	c.3966A>C	p.Arg1322Ser	LP	LP	AD; de novo	[45]
Case 14 (F)	*SCN1A*	c.4784T>C	p.Leu1595Pro	LP	N/A	AD; de novo	N/A
Case 16 (F)	*SCN1A*	c.1214del	p.Leu405Trpfs*10	P	N/A	AD; de novo	N/A
Case 17 (F)	*SCN1A*	c.2855G>A	p.Trp952*	P	P	AD; de novo	[46]
Case 20 (F)	*SCN1A*	c.2415+1delG	canonical splice-site	P	P	AD; de novo	[46]
Case 22 (F)	*SCN1A*	c.4906C>T	p.Arg1636*	P	P	AD; de novo	[47]
Case 24 (M)	*SCN1A*	c.4793A>T	p.Tyr1598Phe	LP	VUS	AD; parental testing unavailable	[48]
Case 25 (F)	*SCN2A*	c.2014G>A	p.Glu672Lys	VUS	N/A	AD; paternal inheritance (father unaffected)	N/A
Case 19 (M)	*SCN8A*	c.4205G>C	p.Gly1402Ala	LP	N/A	AD; maternal inheritance (mother unaffected)	N/A
Case 4 (M)	*KCNQ2*	c.655A>T	p.Lys219*	P	LP	AD; de novo	N/A
Case 30 (F)	*KCNQ5*	c.2066A>G	p.Gln689Arg	VUS	No	AD; paternal inheritance (father unaffected)	N/A
**Synaptic/neuromuscular transmission disorders**							
Case 7 (F)	*SLC5A7*	c.881T>C/c.597+16C>G	p.Ile294Thr/intronic	LP/VUS	LP/VUS	AR; maternal inheritance (c.881T>C) + second variant unresolved	[49]/NA
Case 10 (F)	*PRRT2*	c.649dup	p.Arg217Profs*8	P	P	AD; parental testing unavailable	[50]
**mTOR pathway disorders**							
Case 8 (F)	*TSC1*	c.587C>T	p.Pro196Leu	VUS	VUS	AD; de novo	N/A
Case 23 (M)	*TSC1*	c.1812T>A	p.Tyr604*	P	N/A	AD; paternal inheritance	N/A
Case 28 (M)	*TSC2*	c.4183C>T	p.Gln1395*	P	P	AD; parental testing unavailable	[51]
Case 29 (F)	*TSC2*	c.5024C>T	p.Pro1675Leu	P	P	AD; parental testing unavailable	[52]
Case 18 (M)	*DEPDC5*	c.4503G>T	p.Gln1501His	VUS	N/A	AD; maternal inheritance (mother unaffected)	N/A
**Mitochondrial/metabolic disorders**							
Case 1 (M)	*SLC6A8*	c.1431_1434del	p.Ser479Alafs*31	P	N/A	XLR; de novo	N/A
Case 5 (M)	*ATP7A*	c.3137C>T	p.Thr1046Ile	LP	LP	XL; de novo	[53]
Case 9 (M)	*NDUFV1*	c.365C>T/c.383G>T	p.Pro122Leu/p.Arg128Leu	P/LP	P/LP	AR; compound heterozygous, biparental	[54]/[55]
Case 11 (F)	*IBA57*	c.286T>C/c.667C>G	p.Tyr96His/p.Arg223Gly	P/VUS	P/VUS	AR; maternal inheritance (c.286T>C) + paternal inheritance (c.667C>G)	[56]/N/A
Case 15 (M)	*ECHS1*	c.520G>T/c.4G>C	p.Gly174Cys/p.Ala2Pro	LP/LP	N/A	AR; compound heterozygous, biparental	N/A
Case 31 (M)	*PNPO*	c.673C>T	p.Arg225Cys	P	P	AR; homozygous, biparental	[57]
**Other metabolic/organelle disorders**							
Case 12 (F)	*PEX1*	c.1435_1439dup/c.1670+1G>A	p.Leu480Phefs*4/Splice-site	P/P	P/LP	AR; compound heterozygous, biparental	N/A/[58]
Case 27 (M)	*PIGA*	c.424G>A	p.Ala142Thr	LP	VUS	XLR; de novo	[59]
**Neurodevelopmental/chromatin/transcriptional disorders**							
Case 6 (F)	*MECP2*	c.62+2_62+3del TG	Splice-site	P	P	XLD; paternal inheritance	[60]
Case 13 (F)	*CDKL5*	c.2767_2768insA	p.Gly923Glufs*6	LP	N/A	XLD; de novo	N/A
Case 21 (F)	*SMC1A*	c.1959_1962del	p.Gly655Profs*4	P	N/A	XLD; de novo	N/A
Case 26 (M)	*SCAF4*	c.1280A>T	p.His427Leu	VUS	N/A	AD; maternal inheritance (mother unaffected)	N/A

Five patients had VUS-only findings. These cases were included as candidate genotype–phenotype associations because of phenotype–gene plausibility and absence of a more likely alternative molecular diagnosis, but they were not considered molecularly confirmed diagnoses. VUSs, particularly heterozygous VUS inherited from apparently unaffected parents, are presented as candidate or potentially contributory findings and require additional evidence for reclassification. **Abbreviations:** AD, autosomal dominant; AR, autosomal recessive; XL, X-linked; XLD, X-linked dominant; XLR, X-linked recessive; ACMG, American College of Medical Genetics and Genomics; P, pathogenic; LP, likely pathogenic; VUS, variant of uncertain significance; N/A, not available.

**Table 3:** Selected genotype-linked treatment observations.

Gene	Cases, n	Response Summary
*SCN1A*	8	Response was heterogeneous; most cases showed partial or poor control with polytherapy. One patient improved after stiripentol (Case 14; missense *SCN1A* variant, p.Leu1595Pro), while one patient with a pathogenic nonsense *SCN1A* variant showed worsening on lamotrigine (Case 22; p.Arg1636*).
*PRRT2*	1	Good response to carbamazepine (Case 10).
*PNPO*	1	Initial favorable response to pyridoxine; recurrent febrile episodes were managed with diazepam (Case 31).
*TSC1*, *TSC2*	4	Mostly partial or poor response; persistent seizures despite multiple ASMs were common.
*KCNQ2*, *KCNQ5*	2	Persistent seizures despite multiple ASMs.

Treatment observations were extracted retrospectively from available clinical records and were not based on a standardized treatment response scale. Follow-up duration under specific regimens, seizure frequency diaries, seizure-free intervals, and percentage seizure reductions were not consistently documented; therefore, response categories should be interpreted as qualitative case-level observations rather than comparative efficacy outcomes. **Abbreviations:** ASM, anti-seizure medication.

**Table 4:** *SCN1A* genotype–phenotype spectrum across clinical subgroups.

Clinical Feature	Dravet Syndrome/Dravet-Compatible (n = 2)	Dravet Spectrum/Borderline Dravet (n = 2)	Intermediate *SCN1A*-Related Epilepsy (n = 1)	GEFS+/Non-Dravet *SCN1A*-Related Epilepsy (n = 3)
Case	2, 3	17, 20	14	16, 22, 24
Variants	p.Ser1516*; p.Arg1322Ser	p.Trp952*; c.2415+1delG	p.Leu1595Pro	p.Leu405Trpfs10; p.Arg1636; p.Tyr1598Phe
Variant classes	Nonsense; missense	Nonsense; canonical splice-site	Missense	Frameshift; nonsense; missense
Domain	Inactivation gate of the voltage-gated sodium channel alpha subunits; Ion transport protein	Ion transport protein	Ion transport protein	Ion transport protein
Age at onset, months, median (range)	4 (2–6)	5 (4–6)	9 (single case)	47 (11–72)
Predominant seizure phenotypes	Generalized tonic–clonic seizure; focal motor seizures with myoclonic features	Focal seizures; focal non-motor seizures with tonic–clonic evolution; persistent eyelid myoclonia in one case	Focal motor seizures with versive and clonic features	Generalized tonic–clonic seizures; focal to bilateral tonic-clonic seizures; focal motor seizures
Developmental profile	From no apparent delay at early assessment to severe developmental delay with absent speech and delayed walking	Mild developmental/behavioral concerns to mild developmental delay with speech impairment	Developmental delay with speech impairment; ASD features; motor milestones preserved	Broadly preserved or age-appropriate development
Notable additional features	Pigmentary lesions, restrictive PDA, retinal angiopathy in one case; lower-limb hypotonia in one case	Moderate upper-limb hypotonia in one case	Mild craniofacial dysmorphism	Craniofacial dysmorphism or strabismus in two cases
Overall interpretation	Early-onset *SCN1A*-related epilepsy compatible with classical or near-classical Dravet presentation	Early-onset *SCN1A*-related epilepsy with features bridging Dravet and milder *SCN1A* phenotypes	Intermediate phenotype within the GEFS-Dravet continuum	Later-onset, developmentally milder *SCN1A*-related epilepsy consistent with the non-Dravet spectrum

*SCN1A* subgrouping was descriptive and based on the combined assessment of age at onset, seizure types, febrile/temperature sensitivity where documented, developmental trajectory, EEG features, and treatment course. Dravet syndrome/Dravet-compatible cases were characterized by early onset, multiple or severe seizure types, developmental impairment, and drug-resistant course. Dravet-spectrum/borderline cases showed early onset and Dravet-like features but less complete or less severe developmental/electroclinical expression. The intermediate category was used for cases with developmental or behavioral involvement beyond typical GEFS+ but not fulfilling a classical Dravet-compatible profile. GEFS+/non-Dravet cases were characterized by later onset and/or comparatively preserved development with milder overall clinical course. These categories were used to describe a phenotypic continuum rather than to provide definitive nosologic reassignment. **Abbreviations:** ASD, autism spectrum disorder; GEFS+, genetic epilepsy with febrile seizures plus; PDA, patent ductus arteriosus.

## Data Availability

All clinicogenetic data underlying the descriptive analyses are provided in the Supplementary Materials (Table S1). The raw genomic data supporting the findings of this study are not publicly available because of genetic data privacy considerations and the sensitive nature of genomic information, particularly in a pediatric cohort. Additional de-identified case-level data may be made available from the corresponding author upon reasonable request, subject to institutional and ethical approval and compliance with applicable data protection requirements. Requests should include a brief research proposal, the intended use of the data, and confirmation that the data will be used only for approved research purposes. Data sharing will be limited to variables that cannot reasonably identify individual participants.

## Supplementary Materials

The following supporting information can be downloaded at: https://www.mdpi.com/article/10.3390/jcm15103625/s1, Table S1: Case-level clinicogenetic dataset of the cohort and primer sequences used for Sanger validation. References [44,45,46,47,48,49,50,51,52,53,54,55,57,58,59,60] are cited in the Supplementary Materials.

## Abbreviations

The following abbreviations are used in this manuscript:

ACMG	American College of Medical Genetics and Genomics
AMP	Association for Molecular Pathology
AD	autosomal dominant
AR	autosomal recessive
ASD	autism spectrum disorder
ASM	anti-seizure medication
CNV	copy number variant
EEG	electroencephalography
GEFS+	generalized epilepsy with febrile seizures plus
GRCh38	Genome Reference Consortium Human Build 38
INDEL	insertion/deletion variant
LP	likely pathogenic
MRI	magnetic resonance imaging
PCR	polymerase chain reaction
P	pathogenic
SNV	single-nucleotide variant
VUS	variant of uncertain significance
WES	whole-exome sequencing
XL	X-linked
XLD	X-linked dominant
XLR	X-linked recessive
